# Chromium speciation in water using magnetic polyaniline nanoparticles coupled with microsampling injection-flame atomic absorption spectroscopy

**DOI:** 10.55730/1300-0527.3636

**Published:** 2023-10-29

**Authors:** Osman ÇAYLAK

**Affiliations:** Department of Pharmacy Services, Vocational School of Health, Sivas Cumhuriyet University, Sivas, Turkiye

**Keywords:** Magnetic nanoparticles, Fe_3_O_4_@PANI, water, preconcentration

## Abstract

The chromium speciation procedure was optimized using magnetic polyaniline nanoparticles (Fe_3_O_4_@PANI NPs) solid-phase extraction coupled with microsampling injection-flame atomic absorption spectrometry (MIS-FAAS). Chromium speciation was successfully achieved by Fe_3_O_4_@PANI NPs at pH 8.0. The recoveries obtained for Cr(III) and Cr(VI) were above 95% and under 5%, respectively. Recoveries of over 95% for Cr(III) from 40.0 mL of the sample were obtained using 25 mg Fe_3_O_4_@PANI NPs and 500 μL of 0.2% (w/v) thiourea (TU) solution prepared in 2 mol L^−1^ HCl as eluent. Total chromium as Cr(III) was extracted quantitatively after reducing the Cr(VI) to Cr(III). The linear range, detection limit, preconcentration factor, and precision of the optimized method for Cr(III) in aqueous solution were 2.5–94.0 μg L^−1^, 0.335 μg L^−1^, 80, and 3.07%, respectively. The validation of the method was controlled using SPS-WW2 Batch 114 wastewater and BCR-715 industrial wastewater as standard reference materials (SRMs) for environmental water, and the obtained results were in close agreement with the certified values.

## 1. Introduction

Water pollution caused by heavy metals, pharmaceutical residues, dyes, and pesticides released into surface and groundwaters through industrial, urban, agricultural, and domestic applications has become a significant environmental problem. These pollutants circulate along the food chain and eventually accumulate, creating potential threats to the environment. Especially heavy metals accumulate in the environment in such a way that they cannot be naturally decomposed. These wastes can be dangerous for humans and the ecosystem due to their harmful and carcinogenic nature [1–3].

Chromium is one of the best examples of heavy metals whose different chemical species exhibit opposite chemical and biological behaviors. It consists of oxidation states ranging from −2 to + 6 inclusively. However, trivalent chromium and hexavalent chromium ions, as more stable oxidation states, are found in aquatic environmental samples. In nature, Cr(III) compounds are slightly soluble and comparatively sedentary, but Cr(VI) compounds are more soluble and highly mobile. Cr(III) exists as Cr(OH)^2+^ and Cr(OH)_4_^−^ ions, while Cr(VI) exists as CrO_4_^2−^, HCrO_4_^−^, and Cr_2_O_7_^2−^ anions depending on pH values [4]. The level of toxicity and bioavailability of chromium varies considerably depending upon its oxidation states and concentration [5]. Cr(III), which is necessary for glucose, protein, and lipid metabolisms, is essential for human and animal health [6]. However, Cr(III) may exert a toxic effect on living organisms depending on its concentration. In contrast to Cr(III), Cr(VI) compounds, which have cancerogenic and mutagenic features, are more toxic. The threshold limit concentrations of Cr(III) and Cr(VI) ions in fresh water, irrigation water, and sea water are 50:1, 8:1, and 5:8 μg L^−1^, respectively [7]. According to World Health Organization (WHO) and European-Union Council (EUC) [8,9], the allowed concentration of Cr(VI) in drinking water should be 50 μg L^−1^. The US Environmental-Protection Agency (USEPA) has set a limit of 100 μg L^−1^ for the total chromium concentration in drinking water for human health [10]. However, due to the different chemical and biological properties mentioned above, the determination of total chromium cannot explain its bioavailability and potential human health risks from chromium species in environmental water samples, such as drinking water, hot spring water, seawater, industrial wastewater, etc. To ensure the attainment of this essential information, it is crucial to develop a speciation method with high sensitivity and reliability for the determination of Cr(III) and Cr(VI) in environmental water samples [11]. Additionally, speciation analysis of Cr(III) and Cr(VI) ions is necessary for monitoring environmental pollution, as well as determining the nutritional value and toxicity of foodstuffs. Flame atomic absorption spectrometry (FAAS) is routinely employed for trace metal determination due to its high accuracy and precision, as well as its simplicity and availability. However, FAAS is not selective and sensitive to trace element species and requires pretreatment for the determination of chromium ions. Therefore, there is a need for an enrichment and speciation procedure that distinguishes chemical species. To date, chromatographic or nonchromatographic approaches coupled to element-selective atomic spectroscopic instruments have been used for chromium speciation [5,11–13]. Nonchromatographic techniques such as solid-phase extraction (SPE) [14,15], liquid-liquid extraction (LLE) [16,17], and cloud-point extraction (CPE) [18,19] are performed using simple instruments and offer the capacity to concentrate desired species. Additionally, these techniques are characterized by their simplicity, rapidity, cost-effectiveness, and ease of integration into any analytical chemistry laboratory. Among these extraction methods, magnetic solid phase extraction (MSPE), in which magnetic nanoparticles (MNPs) are used as a solid phase in SPE, has provided several dominant advantages such as lower cost, simplicity, quickness, reduced solvent use, and high enrichment factor (EF) [5,20,21]. The use of magnetite (Fe_3_O_4_) as a magnetic nanoparticle sorbent is more common in MSPE studies due to its relatively high-efficiency synthesis, cheapness, low toxicity, and homogeneous size distribution. However, because of the probable aggregation and oxidation of Fe_3_O_4_ in an aqueous solution, nanoparticles are coated with various substances to develop feasible and effective preconcentration and speciation methods. Among these substances, conductive polymers containing highly delocalized π system with nitrogen, oxygen, and/or sulfur groups that can complex with metal cations are more appropriate options for metal speciation analysis without the need for complexing agents [22,23]. These polymers can constitute different sorption mechanisms, including complexation, ion exchange process, acid-base, and π-π interaction [24]. Since the development of magnetic conductive polymers (MCPs) such as magnetic polythiophene (Fe_3_O_4_@PTh) [25,26] and magnetic polythionine (Fe_3_O_4_@polythionine) [27], they have been investigated for the selective concentration of trace metal ions from complex matrices. Recently, the applications of different polyaniline-modified magnetic materials and adsorbents for the removal of Cr(VI) from aqueous samples have been overviewed [28]. Additionally, some authors have recently published comprehensive papers on the removal of well-known wastes from aqueous media. In one of these studies, Khan et al. encapsulated the ultrafine silico-manganese fumes (SMF) into alginate (cSMFB). It was reported that this encapsulated form exhibits high performance in removing methylene blue (MB), methylene green (MG), crystal violet (CV), and celestine blue (CB) from the aqueous environment [29]. Especially, carbon nanotubes and their modified forms are widely preferred for waste removal. In a recent review article, Alothman and Wabaidur explained the popular application of carbon nanotubes in extraction and chromatographic analysis [30]. As noted, our study also includes a determination procedure. AlFaris et al. introduced an ultrahigh-performance liquid chromatography tandem mass spectrometric method, especially for the determination of aflatoxins [31]. Additionally, the speciation of tellurium and selenium by polyaniline-functionalized MSPE combined with ICP/MS detection [32] and the extractive determination of methylmercury in seawater by GC-MS [33] have been reported. The extractive preconcentration of Cr(VI) by Fe_3_O_4_/polyaniline nanoparticle/HPLC-UV [34] and polyaniline coated magnetic graphene oxide (MGO@PANI)/GFAAS [35] has been studied. However, previous studies [28,34,35] have concluded that the use of Fe_3_O_4_@PANI nanoparticle as adsorbents in MSPE should be thoroughly reconsidered due to inconsistent working pHs. Meanwhile, a combination of the proposed MSPE method, which provides extracts (effluents) in low milliliter volumes (up to 0.5 mL), with MIS-FAAS, requiring a sample volume in the range of 75 to 100 μL for measurement, was also studied to obtain a high preconcentration factor [36]. Previously, no report on the speciation analysis of chromium had been established for the preconcentration of Cr(III) ions using a magnetic PANI coupled with MIS-FAAS. Hence, the use of PANI-coated Fe_3_O_4_ nanoparticles for the preconcentration of Cr(III) and the application of the MIS-FAAS system for speciation are novel. Ultimately, the proposed procedure was employed for chromium speciation and determination in various water samples.

## 2. Experimental

### 2.1. Apparatus

A PerkinElmer AAnalyst700 atomic absorption spectrometer (Norwalk, CT, USA) fitted with a Cr hollow cathode lamp and a handcrafted microsample injection system was used. The parameters for AAS measurement were used as recommended in the instruction manual: wavelength, 357.9 nm; lamp current, 30.0 mA; slit width, 0.7 nm; acetylene flow, 2.0 L min^−1^; and airflow, 17.0 L min^−1^. The MIS provides an appropriate degree of absorbance using a 75–100 μL sample injected with a micropipette into the nebulizer of the spectrometer [36]. A PerkinElmer UATR Series 2 model attenuated total reflection-Fourier transform infrared (ATR-FTIR) spectrometer (PerkinElmer, Waltham, MA, USA) was used to perform the FTIR analysis. A pH meter (WTW/inoLab pH 720, Weilheim, Germany), a heating-controlled magnetic stirrer (J.P. Selecta, Barcelona, Spain), and ultrasonic elution was performed. The ultrasonic elution was conducted using a programmable Bandelin ultrasonic bath (Bandelin Electronic, Berlin, Germany) with a 4 L capacity and a frequency of 35 kHz, allowing adjustment of its temperature in the range of 0 to 80 °C. The ultrapure (UP) water (18.2 MΩ.cm) was obtained using a water purification system (Barnstead, MA, USA). Nuve ST 402 model water bath (Nuve, Industry and Materials Manufacturing and Trade Inc., İstanbul, Türkiye) was used in the adsorption experiments.

### 2.2. Standard solution and reagents

Analytical-grade reagents were used, and solutions were prepared using ultrapure water. Ferric chloride (FeCl_3_.6H_2_O, Shanghai Chemical Reagent Corporation, Shanghai, China), ferrous sulfate (FeSO_4_.7H_2_O, Panreac, Barcelona, Spain), and aniline monomer (C_6_H_7_N) (Merck, Darmstadt, Germany) were used in the synthesis of Fe_3_O_4_-PANI. Hydrochloric acid (37%, v/v), nitric acid (65%, v/v), H_3_PO_4_ (85%, v/v), NaOH, glacial acetic acid, ammonium persulfate (peroxide sulfate) (APS) (NH_4_)_2_S_2_O_8_, ammonia solution (25%, v/v), potassium chloride (KCl), sodium dihydrogen phosphate (NaH_2_PO_4_), and disodium hydrogen phosphate (Na_2_HPO_4_) were obtained from Sigma-Aldrich Company Ltd. (St. Louis, MO, USA). Standard stock solutions of Cr(III) (Sigma-Aldrich) and Cr(VI) (Merck) at 1000.0 mg L^−1^ were used to prepare fresh daily stock standards and calibration standard solutions. A daily freshly prepared aqueous (1 mol L^−1^) hydroxylamine solution (Sigma-Aldrich) was used as a reducing reagent. The desired pH values of the test and sample solutions were performed using a KCl/HCl buffer to pH 2.0, phosphate buffer (H_2_PO_4_^−^/H_3_PO_4_) to pH 3.0, HAc/NaAc buffers to pH 4.0–6.0, with HPO_4_^2−^/HCl buffers to pH 7.0, and ammonia/ammonium chloride buffers to pH 8.0–10.0. Certified reference materials, SPS-WW2 Batch 114 wastewater (Oslo, Norway) and BCR-715 industrial wastewater (LGC, Manchester, USA), were used to verify the accuracy of the proposed procedure.

### 2.3. Preparation of Fe_3_O_4_@PANI

The procedure for the synthesis of Fe_3_O_4_@PANI nanoparticles is schematized in Scheme, which includes two steps: the preparation of Fe_3_O_4_ nanoparticles and their surface modification with PANI. Fe_3_O_4_ nanoparticles were synthesized according to previously established methods [37]. In this experiment, 2.4053 g of FeSO_4_.7H_2_O and 4.72 g of FeCl_3_.6 H_2_O were dissolved in 80.0 mL of water. The solution was stirred for 1 h at 80 °C by adding 10.0 mL of concentrated aqueous ammonia (28% by weight) under a nitrogen atmosphere, using a magnetic stirrer. Then, the Fe_3_O_4_ nanoparticles formed were neutralized by washing with UP water and dispersed in 100.0 mL of UP water.

To modify the surface of Fe_3_O_4_ NPs with PANI, according to the literature reported [38], 4.0 mL aniline and 1.0 mL hydrochloric acid were added to the dispersed Fe_3_O_4_ NPs solution and stirred for 30 min. Afterward, 8.0 mL of 0.1 M (NH_4_)_2_S_2_O_8_ (APS) solution was added dropwise and incubated for 3 h at 0–5 °C. The resulting dark-green Fe_3_O_4_@PANI NPs were gathered using an exterior magnetic field, washed with ethanol and UP water in sequence, and dried for 12 h at 45 °C. The dried Fe_3_O_4_@PANI NPs were characterized by ATR-FTIR spectroscopy.

### 2.4. Procedure for the speciation analysis of chromium

The aim of the study is a simple two-step procedure with a first selective determination of Cr(III), followed by a total chromium determination after the reduction of Cr(VI) to Cr(III). Thus, the MSPE procedure based on Fe_3_O_4_@PANI magnetic nanoparticles was optimized using an aqueous sample solution containing Cr(III) (Scheme). For the determination of Cr(III), 0.5 mL of 3 μg mL^−1^ Cr(III) and Cr(VI) solution taken into a 50.0 mL beaker was diluted to 40.0 mL with UP water and buffered to the desired pHs. After adding 25 mg Fe_3_O_4_@PANI NPs, the solution was immediately shaken manually for 5 min.Fe_3_O_4_@PANI NPs phase loaded with Cr(III) ions was magnetically separated from the aqueous phase by a neodymium magnet, and the supernatant was decanted. Then, 500 μL of 0.2% (w/v) TU solution prepared in 2 mol L^−1^ HCl as eluent was added to the beaker and sonicated for 15 min, for elution of Cr(III) from Fe_3_O_4_@PANI NPs. Finally, the adsorbent was collected on one side of the beaker using an external magnet, and 100 μL fractions of the supernatant (effluent) were injected into the micropipette tip connected to the nebulizer of FAAS for the determination of Cr(III) by MIS-FAAS [36]. The MSPE procedure for total Cr was almost the same as that of Cr(III) after the reduction of Cr(VI) ions to Cr(III) in the sample solution according to the method reported in a previous study [39]. Firstly, 1.0 mL of 2 mol L^−1^ HCl was added, followed by the addition of 1.0 mL of 1 mol L^−1^ hydroxylamine to the solution containing Cr(III) and Cr(VI). The solution was left to reduce Cr(VI) to Cr(III) for at least 20 min at room temperature and then processed for the total chromium determination. The concentration of Cr(VI) was calculated by the difference between total Cr and Cr(III).

### 2.5. Analysis and preparation of real samples

The chromium speciation procedure mentioned above was applied to various water samples, including tap water, pretreated thermal red water, thermal water, Caspian seawater, and Mediterranean coastal seawater samples, collected from Denizli Vocational School of Technical Sciences, Doğa Thermal Health & SPA Hotel’s pretreated red water well, one of travertine thermal pools in Pamukkale, Denizli, Türkiye, Caspian seawater collected from the coast of Sumqayıt city close to Baku, Azerbaijan), and Konyaaltı beach in Antalya, Türkiye, respectively. Using a 0.45 μm nylon membrane filter (Sartorius, Germany), each water sample was filtered, and then they were subjected to the abovedescribed speciation procedure.

The same procedure was also applied to 1:20 diluted SPS-WW2 Batch 114 wastewater and BCR-715 industrial wastewater samples for validation.

## 3. Results and discussion

The efficiency of magnetic PANI for speciation or selective preconcentration of chromium ions could be affected by various parameters, such as sample pH, Fe_3_O_4_@PANI NPs amount, sample volume, eluent type and volume, different elution processes and time, and concentration of coexisting ions. Therefore, the experimental parameters for the purposed MSPE were optimized using a one-variable-at-a-time optimization approach. Following the optimization of the entire procedure, the optimized method was validated concerning different Figures of merit.

### 3.1. Effects of pH

**Figure 1:**
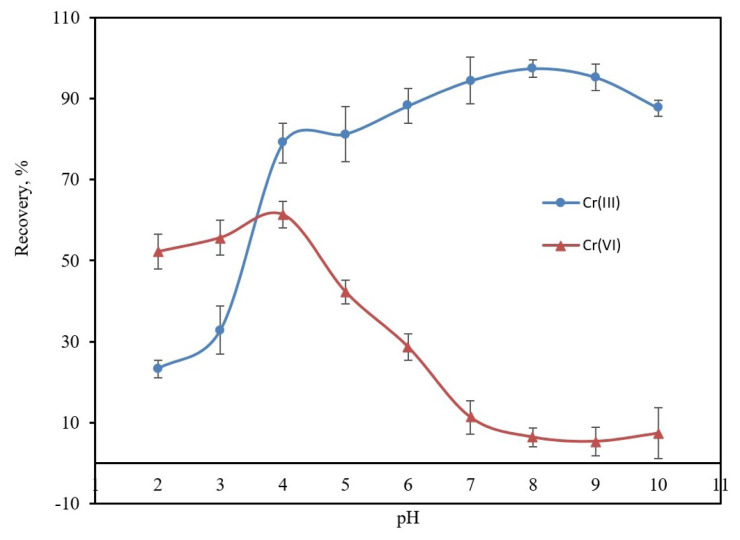
Effect of pH on recovery of chromium ions. Sample volume: 25 mL, concentration of Cr(III) and Cr(VI): 0.5 mg L^−1^; eluent: 5 mL of 0.2% (w/v) thiourea in 2 M HCl; 100 mg Fe_3_O_4_@PANI; n = 3.

Sample pH is critical in the interaction mechanism of Cr(III) and Cr(VI) species with the surface of magnetic PANI NPs, thus affecting extraction efficiency and selectivity. The influence of pH on the adsorption behavior of Cr(III) and Cr(VI) was explored over the pH range of 2.0 to 10.0. It can be seen from Figure 1 that the extraction yield of Cr(III) increases as the pH rises from 2.0 to 6.0 and then reaches almost constant recovery values above 88% in the range of 6.0 to 10.0. The influence of solution pH on Cr(III) recovery could be explained by both the electrostatic interaction and complex formation mechanisms between Cr(III) ions and nitrogen-containing groups (amine and imine) on PANI. The decrease in adsorption capacity at lower pH may be ascribed to proton (H^+^), which compete with Cr(III) ions and occupy the adsorption sites, or it may be due to protonation at the binding sites of the adsorbent [40]. Due to PANI having a pH_pzc_ (pH at the point of zero charge) of about 5.8, the nitrogen atoms of PANI, are protonated in acidic solutions. Therefore, the PANI surfaces carrying a positive charge are not convenient for the adsorption of positively charged Cr(III) species (Cr^3+^, Cr(OH)^2+^, and Cr(OH)_2_^+^ ions) in solutions with pH levels lower than 7.0. This is due to the electrostatic repulsion forces between Cr(III) species and polyaniline positive sites (amine (−NH_2_^+^−) and imine(−NH^+^=)) [4,41]. The increase in the recovery values of Cr(III) ions as the pH increases from 2.0 to 6.0 could be explained by the decrease in the positive charge on the PANI [42]. At pHs > pH_pzc_, the deprotonation of protonated amine (−NH_2_^+^−) and imine(−NH^+^=) groups in the PANI and surface complexation of Cr^3+^ may occur simultaneously on the surface of Fe_3_O_4_@PANI NPs [43]. At the same time, with a further increase in pH, the deprotonated polyaniline gains a partial negative charge (−NHOH−, =NOH−) with increasing hydroxide concentration. Thus, it can be evaluated that the electrostatic attraction forces between this partial negative charge and positively charged Cr(III) ions also contribute to an increase in the recovery values of Cr(III). As a result, the recovery values of Cr(III) increase gradually with the increasing pH of the solution, reaching up to a plateau that includes quantitative recovery values (above 95%) at pH levels in the range of 6.0 to 10.0.

On the other hand, depending on pH, the predominant Cr(VI) species at trace levels in aqueous solutions are HCrO_4_^−^ in the pH range of 2.0 to 6.5 and CrO_4_^2−^ in solutions more alkaline than pH 6.5 [4]. The relatively lower Cr(VI) recovery values (<50%) compared to quantitative recovery (95%) at pH levels lower than pH 6.0 are due to the weaker electrostatic interactions between HCrO_4_^−^ ions and polyaniline positive sites (−NH_2_^+^− and −NH^+^=), as well as an increase in competition between anions from buffers and trace HCrO_4_^−^ ions for anion exchange on the surface of the sorbent [34].

The recovery values of Cr(VI) decrease to 5% at pHs in the range of 6.0 to 10.0 due to the electrostatic repulsion forces between the negatively charged active sites on PANI and CrO_4_^2−^. Based on the data and explanation above, it was concluded that Fe_3_O_4_@PANI NPs provide selectivity between Cr(III) and Cr(VI) ions by adjusting the pH of the sample solution. Therefore, a pH of 8.0 was selected for the speciation of Cr(III) and Cr(VI) species and used in further experiments.

### 3.2. Effect of Fe_3_O_4_@PANI NPs amount and reusability

**Figure 2:**
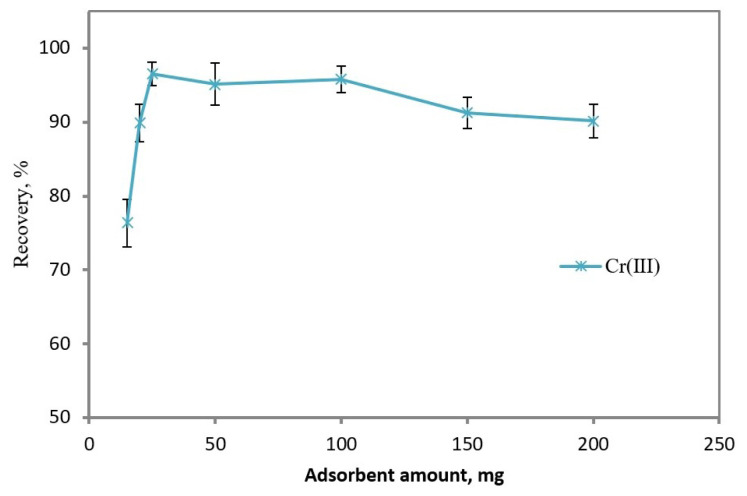
Effect of Fe_3_O_4_/PANI amount on recovery of metal ions. Sample volume: 25 mL, concentration of Cr(III): 0.5 mg L^−1^; eluent: 5 mL of 0.2% (w/v) thiourea in 2 M HCl; pH: 8; n = 3.

An appropriate amount of Fe_3_O_4_@PANI NPs should be used to provide the quantitative extraction of Cr(III), thereby reducing eluent volume and contamination risks from the sorbent. Thus, the influence of Fe_3_O_4_@PANI NPs amount was studied in the range of 15 to 200 mg, and the results were depicted in Figure 2. The extraction capability of Cr(III) improved from 76% to 96% with an increase in the amount of Fe_3_O_4_@PANI NPs from 15 to 25 mg and then almost remained constant. In future studies, 25 mg of Fe_3_O_4_@PANI was preferred as the minimum amount to reduce the risk of possible contamination.

**Figure 3:**
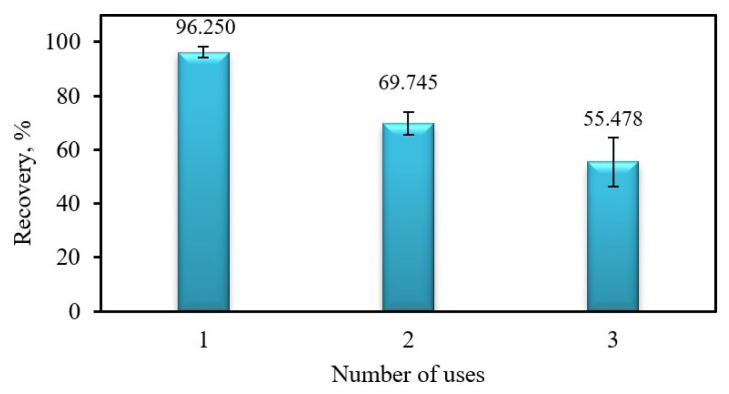
Reusability of Fe_3_O_4_@PANI. Sample volume: 25.0 mL, concentration of Cr(III): 0.5 mg L^−1^; eluent: 5 mL of 0.2% (w/v) thiourea in 2 M HCl; 25 mg Fe_3_O_4_@PANI; pH: 8; n = 3.

To test the reusability of Fe_3_O_4_@PANI NPs, 25 mg of Fe_3_O_4_@PANI NPs were used successively for the general extraction procedure. As can be seen from Figure 3, quantitative recovery was obtained in the first use; however, in subsequent uses, there were significant decreases in the recovery of Cr(III). Presumably, the PANI coated on the Fe_3_O_4_ surface is partially stripped off by the acidic solution used as the eluent in the first adsorption-desorption cycle, resulting in a decline in the analytical performance of Fe_3_O_4_@PANI NPs. It could be concluded from previous studies that this is one of the most important disadvantages of direct polymer coating on the Fe_3_O_4_ surface [22,26,44].

### 3.3. Effect of sample volume, eluent type, and volume

**Figure 4:**
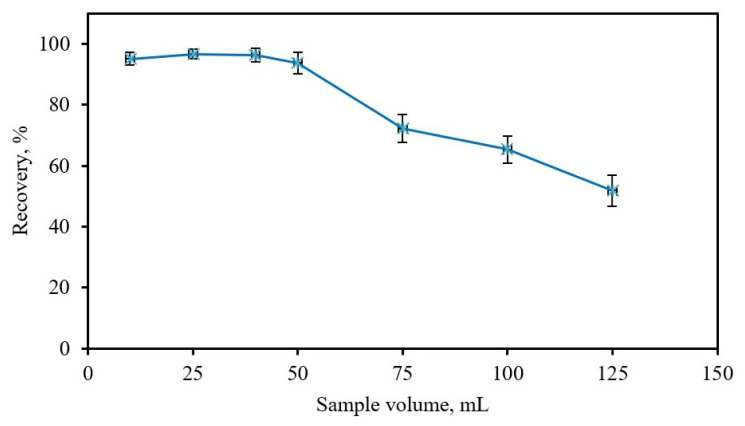
Effect of sample volume on recovery. Concentration of Cr(III): 0.5 mg L^−1^; eluent: 5 mL of 0.2%, w/v thiourea in 2 M HCl; 25 mg Fe_3_O_4_@PANI; pH: 8; n = 3.

To achieve a high concentration factor, the eluent volume should be low, whereas the sample volume should be higher. For this reason, the effect of sample volume on recovery of Cr(III) was studied in the range of 10.0 to 200.0 mL at pH 8.0. As can be seen from Figure 4, it is evident that the recovery of Cr(III) is more than 95% for sample volumes up to 40.0 mL; however, a decrease in recovery (<95%) was observed when sampling volumes exceeded 40.0 mL. Therefore, a sample volume of 40.0 mL was chosen to ensure maximum Cr(III) recoveries.

An appropriate eluent should quantitatively elute analytes from sorbent with as small a volume as possible to achieve a high enrichment factor and provide a suitable medium for the accurate determination of analytes by FAAS [45]. Figure 1 summarizes that Cr(III) adsorbed on the PANI surface would be eluted with an acidic solution. Therefore, firstly, hydrochloric acid and nitric acid solutions were tested as eluent for the elution of Cr(III) from the PANI following the recommended procedure (Table 1). Since it is known that thiourea solutions prepared in the acidic medium are used as eluent in studies with PANI, thiourea solutions prepared with the dilute acid solution were also tested to achieve the recovery values for Cr(III) [46]. The volume of the eluent solution is an important factor for the quantitative recovery of metal ions [47]. The volume effects of the prepared solutions as eluent were evaluated. As seen in Table 1, Cr(III) ions adsorbed on PANI are recovered quantitatively (≥ 95%) using 500 μL of 0.2% (w/v) TU solution prepared in 2 M HCl solution. Therefore, this solution, serving as the eluent, was used in subsequent studies. As a result, the sample volume of 40.0 mL was selected to achieve maximum recoveries of Cr(III), and consequently, the preconcentration factor was calculated as 80, using 500 μL of 0.2% (w/v) TU in 2 M HCl as eluent.

### 3.4. Effects of extraction time

**Figure 5:**
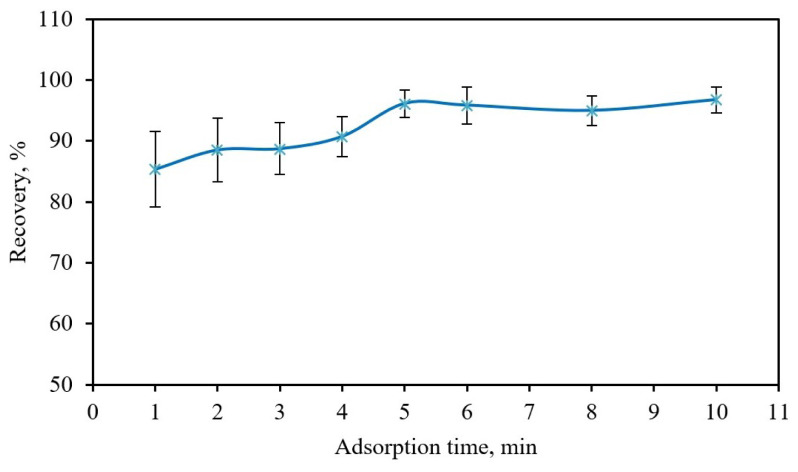
Effect of extraction time on recovery. Sample volume: 40 mL; concentration of Cr(III): 0.5 mg L^−1^; eluent: 0.5 mL of 0.2%, w/v, thiourea in 2 M HCl; 25 mg Fe_3_O_4_@PANI; pH: 8; n = 3.

In the MSPE process, extraction time, defined as the sum of adsorption and desorption times, is one of the significant influencing factors on the extraction efficiency of an analyte. Therefore, the time required for the quantitative adsorption of Cr(III) as an analyte on the Fe_3_O_4_@PANI solid phase was evaluated in the range of 1 to 10 min. For this experiment, 25 mg Fe_3_O_4_@PANI was added to the sample solution buffered to pH 8.0, and the resulting solution was immediately shaken manually. As seen in Figure 5, the highest adsorption efficiency of Cr(III) is reached after 5 min of shaking.

The time required for the quantitative elution of the adsorbed analyte(Cr(III)) from the solid phase into the eluent was also examined. To achieve a short elution time, the elution of Cr(III) adsorbed on the PANI was tested using manual shaking, vortex mixing, and ultrasonic agitation.

As seen in Table 2, quantitative elution of Cr(III) could not be performed within 10 min through manual agitation and vortex mixing. Conversely, the elution efficiency of the analytes increased with prolonged ultrasonication time, reaching quantitative recovery values (≥ 95%) following a 15-min duration. As a result, considering 5 min as the adsorption time of the analyte ions and the 15 min as the elution time, the total extraction time was found to be 20 min.

### 3.5. Effect of coexisting ions

Due to the use of highly selective FAAS in trace metal determinations, possible interferences could be attributed to the preconcentration step of the purposed general procedure. Moreover, the experimental parameters optimized under simple matrix conditions also need to be tested in the presence of possible coexisting ions in real sample matrices. In this context, the influences of possible coexisting ions in natural water samples on the determination of Cr(III) using the proposed procedure were systematically studied (Table S1). The maximum coexisting ion concentration that does not cause an error greater than ± 5% is defined as the tolerable concentration limit for the proposed procedure. Most alkali and alkaline earth metals have less interference than other heavy metals due to their potentially unstable complex formation [48].

The experimental results indicate that in the presence of 3000 mg L^−1^ Na^+^, 1500 mg L^−1^ K^+^, 200 mg L^−1^ Ca^2+^, 100 mg L^−1^ Mg^2+^, 75 mg L^−1^ Cd^2+^ and Co^2+^, 50 mg L^−1^ Mn^2+^ and Ni^2+^, 25 mg L^−1^ Al^3+^, Zn^2+^, Cu^2+^, and Pb^2+^, 5000 mg L^−1^ Cl^−^, 500 mg L^−1^, CH_3_COO^−^, 400 mg L^−1^ SO_4_^2−^, 200 mg L^−1^ CO_3_^2−^, and 25 mg L^−1^ PO_4_^3−^, the recoveries of Cr(III) remained above 90%. Thus, it can be concluded that many coexisting ions have no considerable effect on the preconcentration/determination of Cr(III) in various water samples, and the developed method shows good tolerance to the probable interferences.

### 3.6. Adsorption isotherms

The adsorption characteristics of an adsorbent in SPE studies are defined using various equilibrium adsorption isotherms. The experimental adsorption isotherm of Fe_3_O_4_@PANI nanoparticles for chromium (III) ions was first established using a batch equilibrium technique under optimized conditions [22,49,50]. The sorption equilibrium was investigated with Cr(III) concentrations (C_o_) ranging from 1 to 30 mg L^−1^ in a 50 mL solution containing 10 mg Fe_3_O_4_@PANI nanoparticles, buffered to pH 8.0.

**Figure 6:**
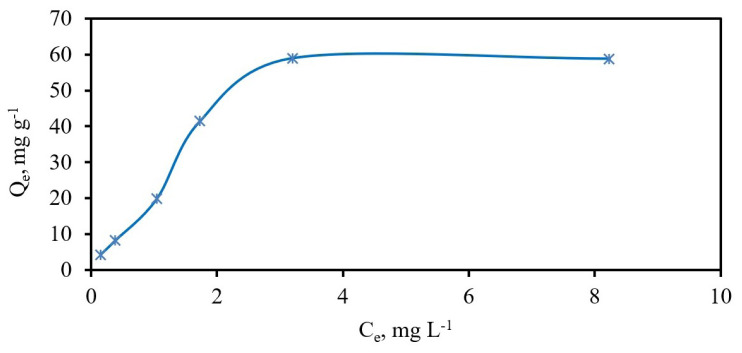
Experimental adsorption isotherm for Cr(III) ions. pH: 8; sample volume: 50.0 mL; 10 mg Fe_3_O_4_@PANI.

By keeping them in a mechanical shaker for 24 h at room conditions (25 ^o^C), equilibrium was achieved between the analyte remaining in the solution and that adsorbed on the solid phase. The solid phase (Fe_3_O_4_@PANI NPs) was then collected utilizing a magnet. The equilibrium concentrations of chromium (C_e_, mg L^−1^) in a clear solution were determined by FAAS. The chromium concentration (Q_e_, mg g^−1^) adsorbed by Fe_3_O_4_@PANI NPs was calculated using the C_o_ and C_e_ concentrations. The experimental isotherm was plotted using the equilibrium concentrations of Cr(III)(Figure 6). The mean adsorption capacity (59.0 mg g^−1^), corresponding to two Q_e_ values on the plateau, was evaluated as experimental adsorption capacity (Q_exp_) for Cr(III). This form of the isotherm, containing a plateau, confirms that the analyte ions are adsorbed as monolayers onto Fe_3_O_4_@PANI NPs [51].

On the other hand, the experimental isotherm data are mostly evaluated using Langmuir and Freundlich isotherms to describe the interaction of an analyte with an adsorbent [44, 49,50,52]. The characteristic Figures obtained from both isotherms applied to experimental data were given in Table 3.

**Figure 7:**
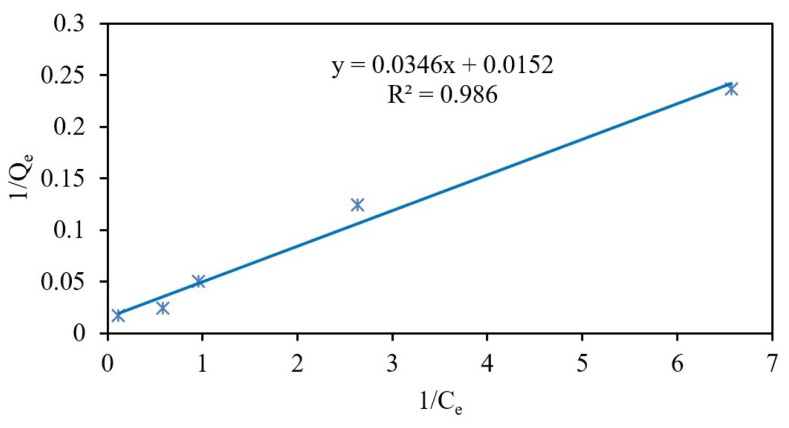
Linearized Langmuir plot for Cr(III) ions.

The linear Langmuir isotherm is formulated as C_e_ / Q_e_ = 1 / (Q_m_ × K_b_) + C_e_ / Q_m_, where C_e_ is the equilibrium concentrations of Cr(III) ions (mg L^−1^) in the solution, Q_e_ is the solute mass adsorbed per unit adsorbent mass at equilibrium (mg g^−1^), K_b_ is the constant of the Langmuir isotherm (L mg^−1^), and Q_m_ is the monolayer adsorption capacity (mg g^−1^) [53]. Langmuir plots of adsorption of Cr(III) on Fe_3_O_4_@PANI were given in Figure 7.

The Langmuir isotherm provides information on the adsorption capabilities of Fe_3_O_4_@PANI. The essential characteristic of a Langmuir isotherm can be expressed in terms of a dimensionless constant separation factor, R_L_, defined by the R_L_ = 1 / (1 + K_b_ C_o_) equation, where C_o_ is the initial concentration, and R_L_ indicates the favorability of a sorption system (R_L_ > 1 unfavorable; R_L_ = 1 linear; 0 < R_L_ < 1 favorable, and R_L_ = 0 irreversible) [44,52–55]. R_L_ values corresponding to initial concentrations Cr(III) in the range of 1 to 30 mg L^−1^ vary from 0.695 to 0.071, indicating favorable chromium adsorption on Fe_3_O_4_@PANI. Additionally, the low K_b_ value found, which is 0.439, confırms a strong binding of Cr(III) ions on Fe_3_O_4_@PANI [52]. The compatibility of this adsorption system is also supported by the maximum equilibrium adsorption capacity (Q_m_, 65.8 mg g^−1^), which is close to the experimental adsorption capacity (Q_exp_, 59.0 mg g^−1^) with a high R^2^ value (Table 3).

**Figure 8:**
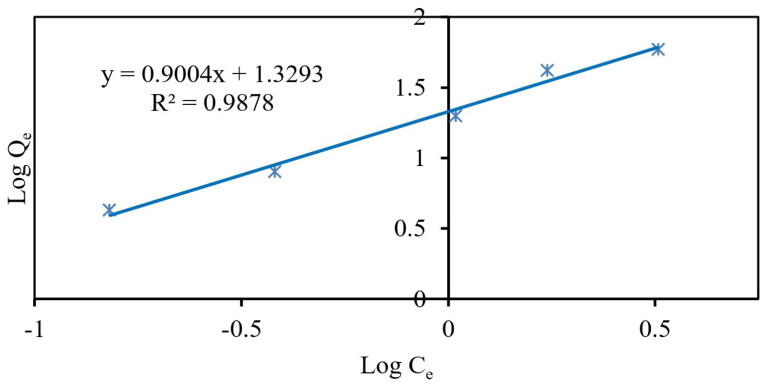
Linearized Freundlich plot for Cr(III) ions.

The linearized Freundlich isotherm equation may be written as lnQ_e_ = lnK_f_ + (1 / n)lnC_e_, where K_f_ and 1/n are Freundlich constants that correspond to the resin adsorption capacity and adsorption intensity of the adsorbent, respectively. As seen in Figure 8, the compatibility of Fe_3_O_4_@PANI with the Freundlich isotherm for adsorption of Cr(III) ions may be explained by the high correlation coefficient (R^2^) found to be 0.988 (Table 3). K_f_ corresponding to the adsorption capacity and adsorption intensity (1/n) for Cr(III) ions was calculated as 21.345 L/g and as 0.900, respectively. A small value of 1/n indicates a stronger interaction between Fe_3_O_4_@PANI as adsorbent and Cr(III) as the analyte, while 1/n close to 1 indicates linear adsorption, leading to the same adsorption energies for all sites [56]. The high correlation coefficient (0.988) indicates that the adsorption is appropriate. Additionally, the value of 1/n < 1 and the greater value of K_f_ define that Cr(III) at low concentrations is affirmatively adsorbed by Fe_3_O_4_@PANI [55].

### 3.7. Analytical characteristics

The analytical data characteristics of the developed MSPE process, combined with MIS–FAAS, were evaluated under the optimal conditions described above (Table S2). The calibration curve was constructed based on the determination of Cr(III) using the general procedure, and good linearity with a correlation coefficient of 0.9994 was obtained between 2.5 and 94.0 μg L^−1^ in 40.0 mL samples. The regression equation was A = 0.6665[Cr(III)] + 0.0018. The calibration curve established using external calibration standards was linear between 0.2 and 7.5 mg L^−1^ and its regression equation was found as A = 0.0086[Cr(III)] + 0.002 with a correlation coefficient of 0.9992. The closeness of PF (80) to the enhancement factor (77.5), which is calculated as the slope ratio of both regression equations, confirms the accuracy of the method. The recovery value (96.4%) of Cr(III), computed as the ratio of PF to EF, is consistent with the experimental recovery of 96.3 ± 2.1% at a 95% confidence level (n = 10). The relative standard deviation (RSD) was 3.1%. This compatibility confirms the accuracy and precision of the method, as well. However, the accuracy of the developed method was also tested by applying the procedure to SPS-WW2 Batch 114 wastewater and BCR-715 industrial wastewater samples as certified reference materials (CRM) (Table 4) and the analyte spiked real samples (Tables 5 and 6). The results obtained were compared with the certificate values at the 95% confidence level using t-test. Since the experimental t-values were lower than the value of t_critical_ = 4.303, it was concluded that there was no difference between the compared values. In addition, the values found in the analyzes of certified reference materials were evaluated according to the procedure described by Linsinger [57]. It was observed that the measured values were not significantly different from the certified value at a 95% confidence level (U_Δ_ ≥ Δ_m_). The relative error is at a maximum −6.7%. The results found are in reasonable agreement with the certified values of CRMs. As a result, it was deduced that the suggested process is feasible for various real water samples. The limit of detection was 0.335 μg L^−1^, and the limit of quantitation was 2.037 μg L^−1^ for Cr(III) at the 99.7% confidence interval according to IUPAC recommendations [58,59].

### 3.8. Applications

The analytical performance Figures show the potential of Fe_3_O_4_@PANI NPs for chromium speciation analysis. To prove this fact, the suggested process was applied to tap water, seawater, and thermal water samples by analyte spiked-recovery experiments (Table 5). The recovery values over 95% for both chromium ions verified that the method is applicable to the speciation of Cr(III) and Cr(VI) ions. The concentrations of both chromium ions in the samples were lower than the quantitation limit of the method.

### 3.9. Comparison with some reported approaches

The analytical performance Figures achieved in this study, as well as those reported in several recent studies on chromium speciation analysis [23,35,60–63], are compared in Table 6. It could be concluded that the procedure exhibits fairly comparable characteristics across various parameters, including capacity, sorbent amount, preconcentration factor, LOD, and RSD when compared with other reported procedures based on the use of sorbent rich in imine, amine, or nitrogen groups. The LOD value was improved by comparing direct FAAS measurements. Another advance of Fe_3_O_4_@PANI NPs is their capability to separate and preconcentrate Cr(III) ions at trace levels in the presence of Cr(VI) and other divers ions in the waters samples, due to ion exchange and complexing mechanisms.

## 4. Conclusion

In this study, the use of magnetic polyaniline nanoparticles-based MSPE combined with MIS-FAAS was evaluated for the first time for the speciation analysis of trivalent and hexavalent chromium ions, providing the preconcentration of Cr(III) at pH 8. It has been determined that the adsorption of Cr(III) is a physicochemical process involving both the electrostatic interactions and complex forming mechanisms between Cr(III) species and nitrogen-containing groups on the PANI. The compatibility of Fe_3_O_4_@PANI NPs with the Langmuir and Freundlich isotherms for the adsorption of Cr(III) ions was explained by high correlation coefficients. Thus, both isotherms support that the adsorption of Cr(III) was a favorable process. Acceptable results were obtained in the application of speciation analysis chromium in environmental water samples. The applicability of chromium speciation at ppb levels makes Fe_3_O_4_@PANI NPs an efficient sorbent for Cr(III).

## Supplementary data

**Table S1:** Effect of coexisting ions on recovery of Cr(III) ions, obtained by the proposed procedure.

Coexisting ions	Added as	Tolerance limit (mg L ^−1^)	Recovery (%)
Na ^+^	NaCl	3000	93 ± 2 ^a^
K ^+^	KCl	1500	93 ± 3
Ca ^2+^	Ca(NO _3_) _2_.2H _2_O	200	91 ± 2
Mg ^2+^	MgSO _4_	100	92 ± 3
Al ^3+^	Al _2_(SO _4_) _3_	25	89 ± 2
Cd ^2+^	Cd(NO _3_) _2_.4H _2_O	75	92 ± 2
Co ^2+^	Co(NO _3_) _2_.6H _2_O	75	93 ± 2
Mn ^2+^	MnSO _4_.H _2_O	50	89 ± 2
Ni ^2+^	Ni(NO _3_) _2_.6H _2_O	50	90 ± 2
Zn ^2+^	Zn(NO _3_) _2_.6H _2_O	25	90 ± 2
Pb ^2+^	Pb(NO _3_) _2_	25	94 ± 2
Cu ^2+^	CuCl _2_.2H _2_O	25	90 ± 2
Cl ^−^	NaCl	5000	92 ± 1
CH _3_COO ^−^	CH _3_COONa.3H _2_O	500	92 ± 2
SO _4_ ^2−^	MgSO _4_	400	91 ± 2
CO _3_ ^2−^	Na _2_CO _3_	200	93 ± 3
PO _4_ ^3−^	Na _3_PO _4_	25	91 ± 3

a Average of three measurements ± standard deviation.

**Table S2:** Analytical performance Figures of the proposed MSPE method.

Analytical performance characteristics	Analytical Figures for Cr (III)
Calibration equation with preconcentration	A = 0.6665[Cr(III)] + 0.0018 / R ^2^ = 0.9994
Calibration equation without preconcentration	A = 0.0086[Cr(III)] + 0.002 / R ^2^ = 0.9993
Linear ranges, mg L ^−1^	0.0025–0.0938/0.2–7.5
Recovery, %	96.3 ± 2.1
Enhancement factor, EF	77.5
Theoretical preconcentration factor, PF	80
Relative error (E _r_, %)	−3.13
Limit of detection (LOD, μg L ^−1^, 3ρ, n = 10)	0.335
Limit of quantification (LOQ, μg L ^−1^, 10ρ, n = 10)	2.037

## Figures and Tables

**Scheme:**
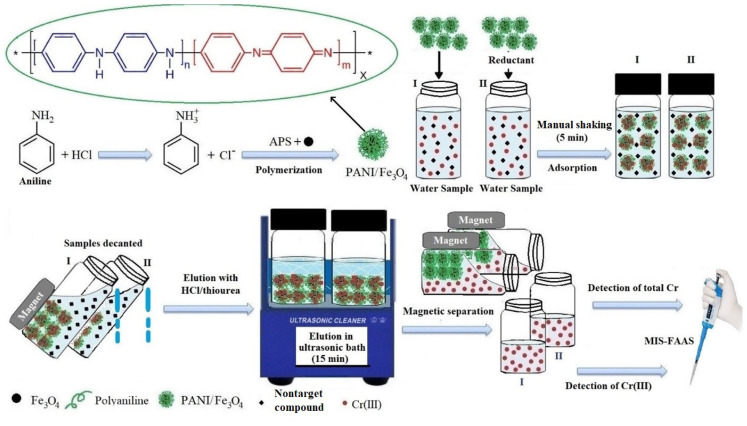
A schematic model for modifying Fe3O4 nanoparticles with PANI and chromium speciation analysis using Fe3O4@PANI NPs coupled with MIS–FAAS.

**Table 1:** Effect of type, concentration, and volume of eluent solution on recovery of analytes. Sample volume: 40.0 mL; concentration of Cr(III): 0.5 mg L^−1^; 25 mg Fe_3_O_4_@PANI; pH: 8; n = 3.

Eluent	Recovery (%)
10.0 mL 1 M HCl	63.2 ± 3.1^a^
10.0 mL 2 M HCl	74.6 ± 1.1
10.0 mL 3 M HCl	76.4 ± 3.2
10.0 mL 1 M HNO_3_	36.2 ± 3.4
10.0 mL 2 M HNO_3_	47.7 ± 3.8
10.0 mL of 0.1%, w/v, thiourea in 2 M HCl	88.2 ± 4.1
10.0 mL of 0.2%, w/v, thiourea in 2 M HCl	95.2 ± 3.3
10.0 mL of 0.3%, w/v, thiourea in 2 M HCl	92.4 ± 3.4
5.0 mL of 0.2%, w/v, thiourea in 2 M HCl	96.4 ± 3.1
2.0 mL of 0.2%, w/v, thiourea in 2 M HCl	94.2 ± 2.2
1.0 mL of 0.2%, w/v, thiourea in 2 M HCl	97.1 ± 3.3
0.5 mL of 0.2%, w/v, thiourea in 2 M HCl	96.3 ± 2.1
0.4 mL of 0.2%, w/v, thiourea in 2 M HCl	90.1 ± 2.9
10.0 mL of 0.1%, w/v, thiourea in 3 M HCl	89.4 ± 3.3

a Average of three measurements ± standard deviation.

**Table 2:** Effect of elution times on recovery of analytes by manual shaking, vortex mixing and ultrasonic agitation techniques. Sample volume: 40.0 mL, concentrations of Cr(III) and Cr(VI): 0.5 mg L^−1^; eluent: 0.5 mL of 0.2%, w/v thiourea in 2 M HCl; 25 mg Fe_3_O_4_@PANI; pH: 8; n = 3.

Elution process	Time, min	Recovery (%)
Manual shaking	5	46.4 ± 3.8 a
	10	48.8 ± 4.3
Vortex mixing,1600 rpm	5	52.4 ± 2.9
	10	59.4 ± 3.2
	5	60.9 ± 3.3
	10	79.4 ± 2.4
Ultrasonic agitation	12.5	88.8 ± 3.0
	15	96.2 ± 2.1
	20	95.3 ± 2.3

a Average of three measurements ± standard deviation.

**Table 3:** The characteristic Figures calculated from experimental, Langmuir, and Freundlich isotherms for the adsorption of Cr(III) ions on Fe_3_O_4_@PANI.

Isotherms	Parameters	Cr(III)
Experimental	Q_m_, mg g^−1^	59.0
Langmuir	Q_m_, mg g^−1^	65.79
	K_b_, L mg^−1^	0.439
	R_L,min–max_	0.695 to 0.071
	R^2^	0.986
	Equation	1/Q_e_ = 0.0346/C_e_) + 0.0152
Freundlich	K_f_, L mg^−1^	21.345
	1/n	0.900
	R^2^	0.988
	Equation	Log Q_e_ = 0.9004LogC_e_ + 1.3293

**Table 4:** The level of total chromium in certified reference materials application of the presented procedure. Certified sample volume: 40 mL; eluent: 0.5 mL of 0.2% (w/v) thiourea in 2 M HCl; 25 mg Fe_3_O_4_@PANI; pH: 8; n = 3.

Certified reference materials	SPS-WW2 Batch 114 wastewater	BCR-715 industrial wastewater
Certified concentration, μg L^−1^	1000 ± 5^a^	1000 ± 90
Obtained concentration, μg L^−1^	958 ± 20	944 ± 43
Error, %	−4.20	−5.56
RSD, %	2.08	4.59
Recovery, %	95.8 ± 2	94.4 ± 4
Value of t_test_^b^	−3.65	−2.22
u_CRM_	2.5	45
u_m_	11.55	74.48
Δ_m_	42	56
u_Δ_	11.82	87.02
U_Δ_ = 2 u_Δ_	23.64	174.04

a Total chromium concentration mean ± standard deviation,

b t-test (n = 3) at 95% confidence level, tcritical = 4.303,

Δm: uncertainty of the measurement result, UΔ: combined uncertainty of result and certified value.

**Table 5:** Analysis of real water samples for chromium speciation. Sample volume: 40 mL; eluent: 0.5 mL of 0.2% (w/v) thiourea in 2 M HCl; 25 mg Fe_3_O_4_@PANI; pH: 8; n = 3.

Samples	Added, μg L^−1^		Found^a^, μg L^−1^			Recovery, %		

	Cr(III)	Cr(VI)	Cr(III)	Cr(VI)^b^	Cr_T_^c^	Cr(III)	Cr(VI)	Cr_T_
Tap water	0	0	BQL	BQL	BQL	-	-	-
	5	5	4.85 ± 0.22	5.35 ± 0.44	10.20 ± 0.31	97 ± 4	107 ± 9	102 ± 3
	10	10	9.44 ± 0.51	10.59 ± 0.88	20.08 ± 0.98	94 ± 5	106 ± 9	100 ± 4
Mediterranean Konyaaltı sea water	0	0	3.17 ± 0.24			-		
	5	5	7.97 ± 0.41			95		
	10	10	12.55 ± 0.51			93		
Caspian Sea water	0	0	BQL	BQL	BQL	-	-	-
	5	5	5.29 ± 0.30	4.94 ± 0.25	10.23 ± 0.28	106 ± 6	99 ± 5	102 ± 3
	10	10	9.96 ± 0.30	10.08 ± 0.89	20.09 ± 0.98	99 ± 3	101 ± 9	100 ± 4
Karahayıt hot spring water (red water)	0	0	BQL	BQL	BQL	-	-	-
	5	5	5.41 ± 0.12	4.88 ± 0.26	10.29 ± 0.22	108 ± 2	98 ± 5	103 ± 2
	10	10	10.49 ± 0.17	10.34 ± 0.63	20.87 ± 0.79	105 ± 2	103 ± 6	104 ± 4
Pamukkale thermal water	0	0	BQL			-		
	5	5	4.74 ± 0.28			94.8 ± 2		
	10	10	9.45 ± 0.44			94.5 ± 2		

BQL: below quantitation limit,

a average value ± standard deviation,

b Cr(VI): concentration of Cr(VI) ions founded by subtracting Cr(III) concentration from Cr_T_ concentration_,_

c Cr_T_: determined after reducing Cr(VI) to Cr (III) ions in sample solutions.

**Table 6:** Comparison of magnetic nanoparticles containing imine and/or amine groups, or nitrogen, coupled with FAAS for chromium speciation analysis.

Magnetic nanoparticles	Analyte ions	Capacity, mg/g	Sorbent amount, mg	PF	LOD, μg/L	RSD, %	References
Fe_3_O_4_@coPANI - PTh	Cr(III)	-	100	40	1.5	1.85–3.4	[23]
MGO@PANI (GFAAS)	Cr(VI)	14.765	40	40	0.005	5.3	[35]
Amino-functionalized Fe_3_O_4_/SiO_2_	Cr(VI)	-	25	16	1.1	3.7	[60]
Trien functionalized magnetite GO (mf-GO)	Cr(III)	9.6	50	10	1.6	3.36	[61]
	Cr(VI)	16.4	50	10	1.4	2.99	
Silica - Fe_3_O_4_− zincon	Cr(III)	9.16	20	100	0.016	6.0	[62]
Fe_3_O_4_-CNT@ dipyridylamine	Cr(III)	215		182	0.5	4.9	[63]
Fe_3_O_4_@PANI	Cr(III)	59.0	25	80	0.335	3.07	This study
